# Utero-ovarian low grade endometrial stromal sarcoma, a case report

**DOI:** 10.1016/j.ijscr.2023.108296

**Published:** 2023-05-04

**Authors:** Mahsa Salehi, Somayeh Sheidaei, Hamid Reza Jafari, Afrooz Jafari

**Affiliations:** a Mazandaran University of Medical Sciences, Mazandaran, Iran; b Isfahan University of Medical Sciences, Isfahan, Iran; c Shahrekord University of Medical Sciences, Shahrekord, Iran

**Keywords:** Endometrial stromal sarcoma, Bilateral ovarian metastasis, Low grade endometrial stromal sarcoma (LG-ESS), Incidental

## Abstract

**Introduction:**

Endometrial stromal sarcoma (ESS) is a rare malignancy of uterine in middle aged women. There are numerous subtypes for ESS which share the same clinical picture of uterine bleeding and pelvic pain. Consequently, diagnosis and treatment modalities of LG-ESS with metastasis are challenging. However, both molecular and immunological study of samples can be useful.

**Case presentation:**

In this case study, we report a 52-year-old woman presenting with the chief complaint of unusual uterine bleeding. There was no specific finding in her past medical history. The CT study revealed enlarged bilateral ovary with a significantly large left ovarian mass and suspicious mass in uterus. By the diagnosis of ovarian mass, patient went under total abdominal hysterectomy with bilateral salpingo-oophorectomy (BSO), greater omentectomy, and appendectomy followed by post-op hormone therapy. Her follow-up was uneventful. The IHC and pathological study of samples revealed incidental LG-ESS uterus mass with metastasis to ovaries despite her primary diagnosis.

**Discussion:**

LG-ESS has low metastasis rate. Surgical modalities and neoadjuvant therapies are recommended base on the stage of ESS. In the following study, we represent a case of incidental LG-ESS with bilateral ovarian invasion who was initially diagnosed as an ovarian mass.

**Conclusion:**

Our patient was successfully managed by surgical intervention. Despite scarcity of LG-ESS, it is advised to consider LG-ESS as a differential diagnosis in management of patients with a uterus mass with bilateral ovarian involvement.

## Highlights

• LG-ESS of uterus
• Bilateral ovarian metastasis of LG-ESS
• Incidental LG-ESS
• Surgical management of LG-ESS

## 1 Introduction

Endometrial stromal sarcoma (ESS) is a rare malignant stromal cell tumor that originates from endometrial stromal cells, and it accounts for approximately 1 % of uterine malignancies and <10 % of uterine stromal neoplasm [1]. Regarding WHO 2020 classification ESS is classified into 4 types which are endometrial stromal nodule (ESN), low-grade endometrial stromal sarcoma (LG-ESS), high-grade endometrial stromal sarcoma (HG-ESS), undifferentiated uterine sarcoma (UUS). LG-ESSs are low grade sarcomas with rare metastatic potential. They have the potential to invade myometrium or vascular structures which differentiates them from ESN. The usual age group of patients is between 40 and 50 years. Considering the clinical picture of all these entities including LG-ESS, they usually cause pathological uterine bleeding, dysmenorrhea, and pelvic pain. LG-ESS has low metastatic rate. There are findings of LG-ESS metastasis to ovaries, vagina, vulva, and abdominal cavity. Pre-operative differential diagnosis includes fibroid uterus, endometrial carcinoma and sarcoma. Despite some contradictory case reports, lymph node metastasis is correlated with poor prognosis [2], [3].

Histologically, ESS reveals closely packed monotonous oval to spindle cells (similar to endometrial stroma) as well as small arteriolar vessels surrounded by such atypical cells. ESN is introduced as endolymphatic stromal myosis and ESS as a stromal sarcoma [4], [5]. LG_ESS has infiltrative pattern with angiolymphatic invasion [6].

Low-grade ESS (LG-ESS) and high-grade ESS (HG-ESS) have different treatments in which the second one has a poor prognosis and low survival rate [1]. Meanwhile, due to rarity of LG-ESS and small sample size of clinical studies on LG-ESS reliable evaluation of prognosis is critical [7]. Cytoreduction is used in tumors with extra-uterine appearance to improve prognosis and life quality [8]. Although BSO (bilateral salpingo-oophorectomy) in reproductive women as a favored treatment has been discussed, it is not a gold standard treatment in perimenopausal and postmenopausal women. Lymphadenectomy for ESS is not recommended unless there is lymph node involvement or extrauterine invasion. Furthermore, other studies also recommend typical hormone therapy including aromatase inhibitors and progestins [8]. Here, we report an incidental challenging case of LG-ESS of uterus with bilateral ovarian metastasis who was presented primarily as an ovarian mass.

## 2 Case presentation

A 52-year-old non-smoker multiparous woman was referred to our hospital by one month history of post-menopausal vaginal bleeding. She has had a normal menstruation cycle for 20 years. At the time of admission, she had stable vital sign and complained of no abdominal symptoms. She did not complain of any irregularities in her bowel habits, fever, and weight loss. Furthermore, she reported no history of contraceptive usage and no alarming findings were detected in her medical history. She had no previous history of diseases or malignancies in her family history. During the last 6 months, she had menorrhagia and metrorrhagia with intermittent abdominal discomfort. Nonetheless, she had not sought medical care due to her poor socio-economic status. However, she was referred to our surgical department based on her imaging. In her physical exam, she had BMI of 27 and she was pale. The abdomen had obese appearance and shape and a mass could be palpated in the left lower quadrant. No lymphadenopathy was detected, as well. Lab data showed anemia of Hb = 10 g/dL. Also, CA-125 was checked which was 45 units/mL.

**Fig. 1:**
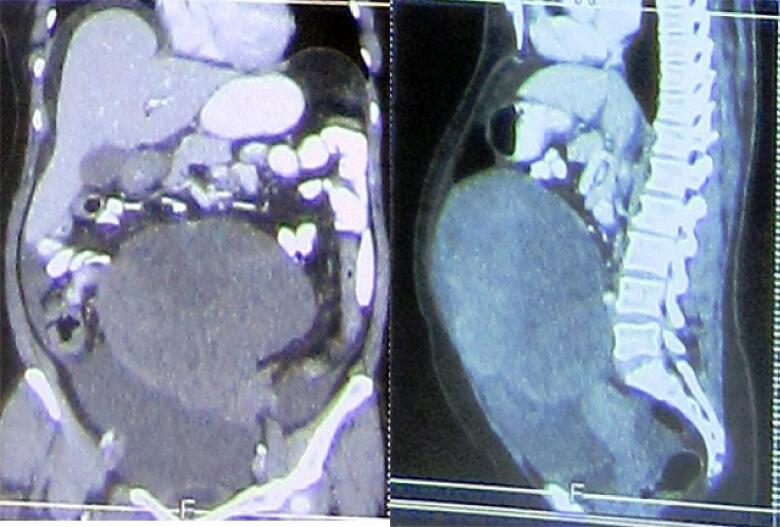
Abdomino-pelvic CT scan, longitudinal and axial view.

**Fig. 2:**
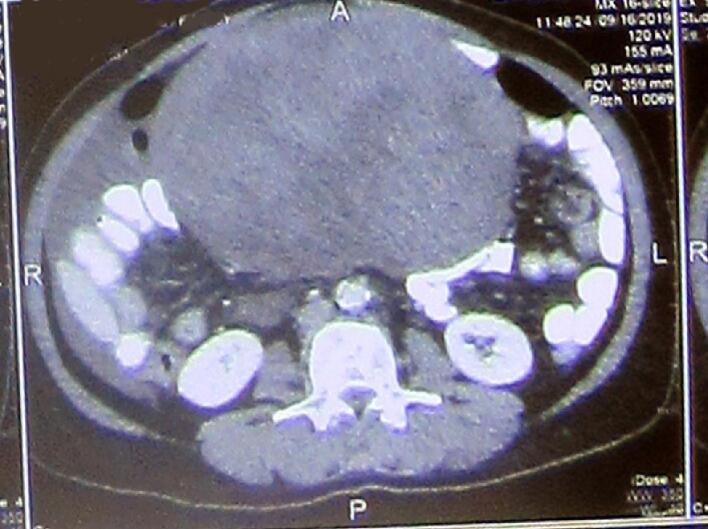
Abdomino-pelvic CT scan, horizontal view.

In regard to imaging, pelvic ultrasonography revealed large left ovarian mass of 15 cm. The abdomino-pelvic CT scan revealed enlarged bilateral ovaries with a large mass of 18 to 6 cm in the left ovary, intra-myometrium necrotic mass with irregular margins, and pelvic tissue reaction with mild transudate in the right iliac fossa. There were no enlarged lymph nodes (Fig. 1, Fig. 2).

Considering the CT scan and clinical findings and the age of our patient, a decision was made to move toward early surgical intervention. In the operating room, primary diagnostic laparoscopy was done which revealed a large left ovarian mass with no macroscopic findings of abdomino-pelvic endometriosis. During laparotomy, there was mild serous fluid collection in the pelvic cavity. There was no sign of metastasis in the peritoneal region of the abdomen and pelvic area. Also, liver, omentum, and bowels were normal. From the carful investigation of the uterus and ovaries, we detected some irregularities and enlargement in both ovarian surfaces. Also, there was a large polypoid ovarian mass in the left lower abdomen with no adhesion or invasion to surrounding pelvic area. Uterus had slightly larger size and no palpable mass could be touched. No lymphadenopathy was detected in the mesothelium of bowels or retroperitoneal region. Hence, a total abdominal hysterectomy with bilateral salpingo-oophorectomy (BSO), greater omentectomy, and appendectomy were done. There was no significant blood loss during the procedure and the patient experienced good recovery in the ward.

**Fig. 3:**
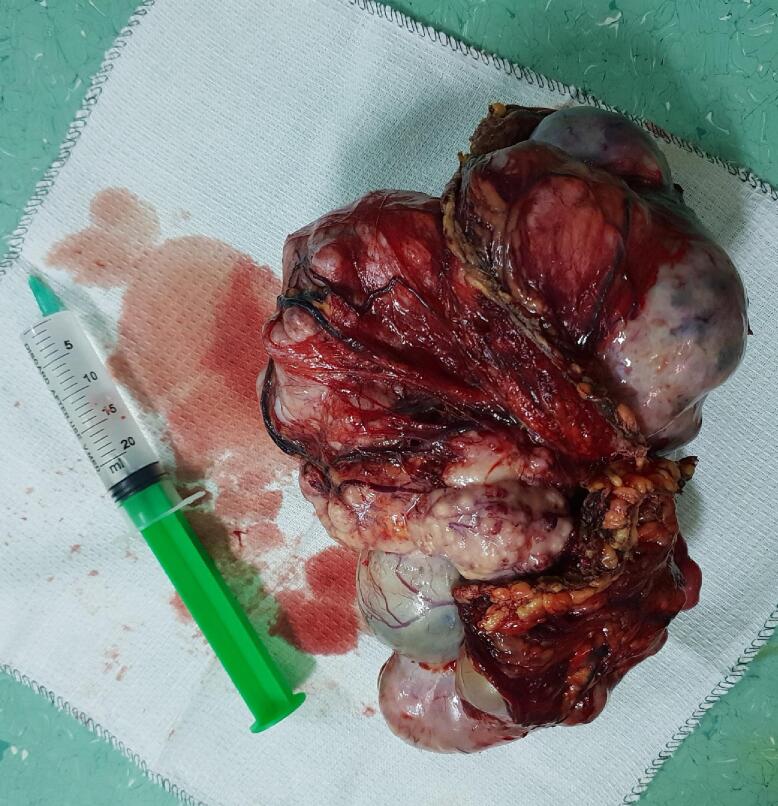
Left ovarian mass.

**Fig. 4:**
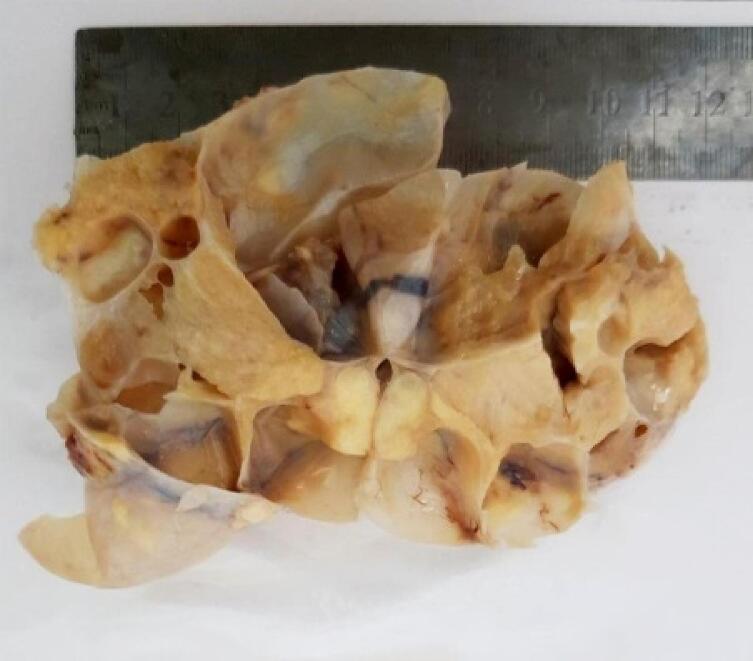
Macroscopic view of ovary.

**Fig. 5:**
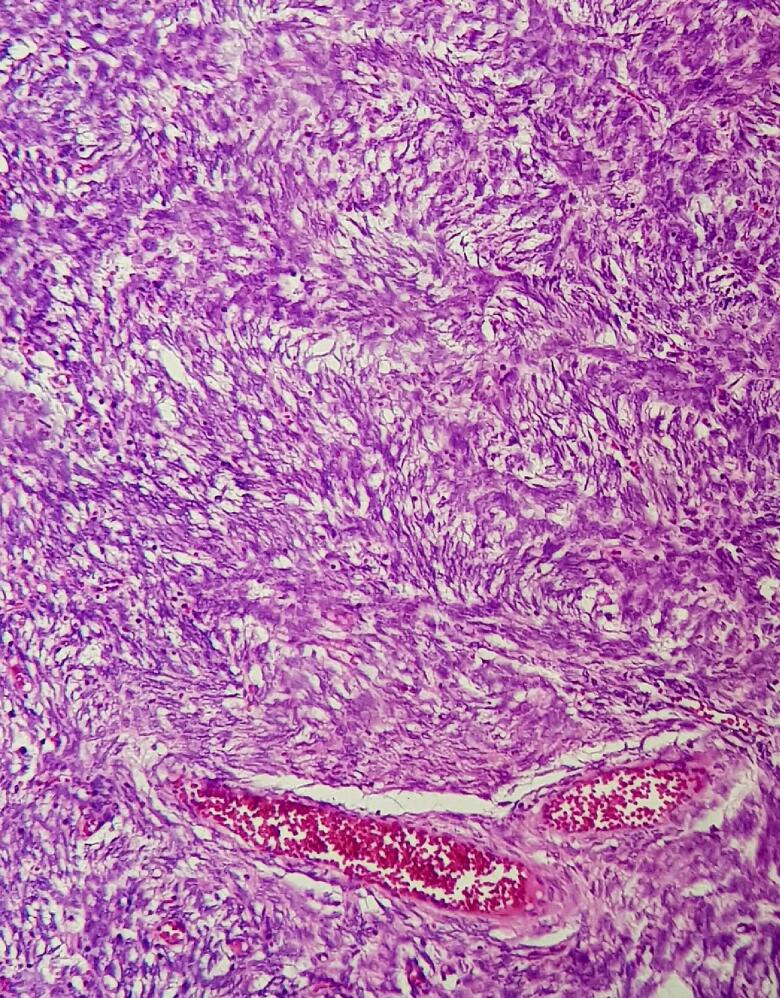
Sheets of monotonous oval to spindle cells with minimal cytologic atypia, vesicular chromatin and scant cytoplasm with low mitotic count and no necrosis in ovary.

Macroscopically, the ovarian mass was 18 to 7 cm with a solid cystic irregular appearance (Fig. 3, Fig. 4). Sections of samples from both ovaries had sheets of monotonous oval to spindle cells with minimal cytologic atypia, vesicular chromatin, and scant cytoplasm (Fig. 5).

**Fig. 6:**
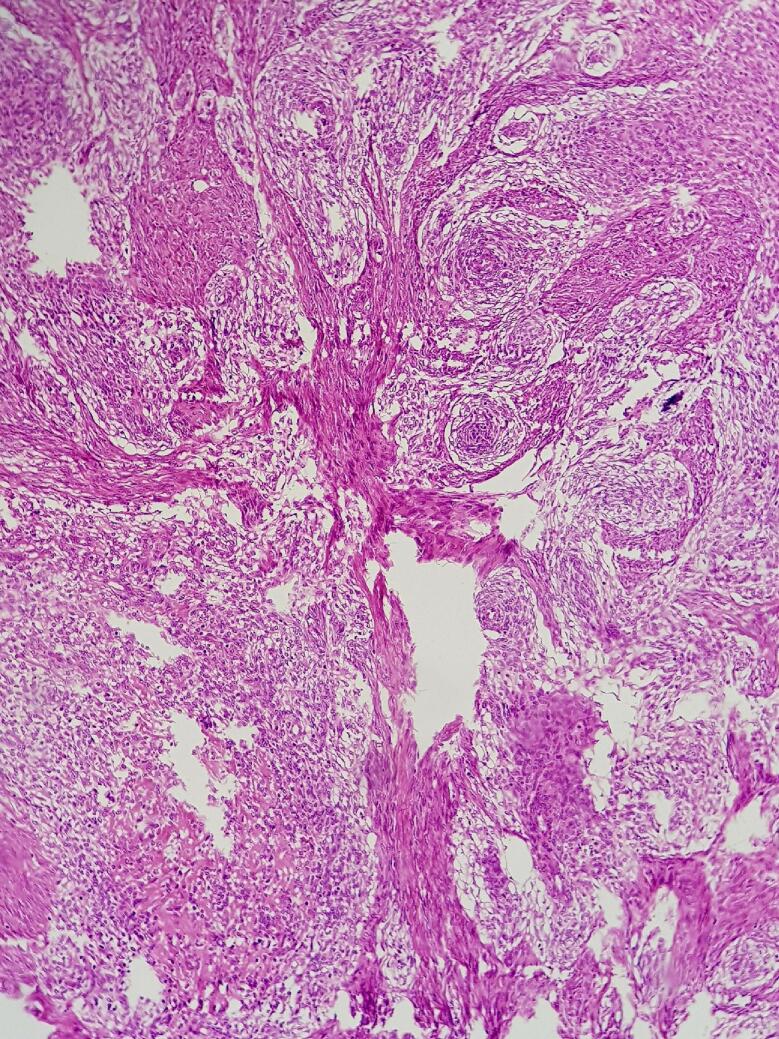
Irregular cellular islands, forming permeative tongue-like pattern of myometrial invasion with low mitotic count and no necrosis.

**Fig. 7:**
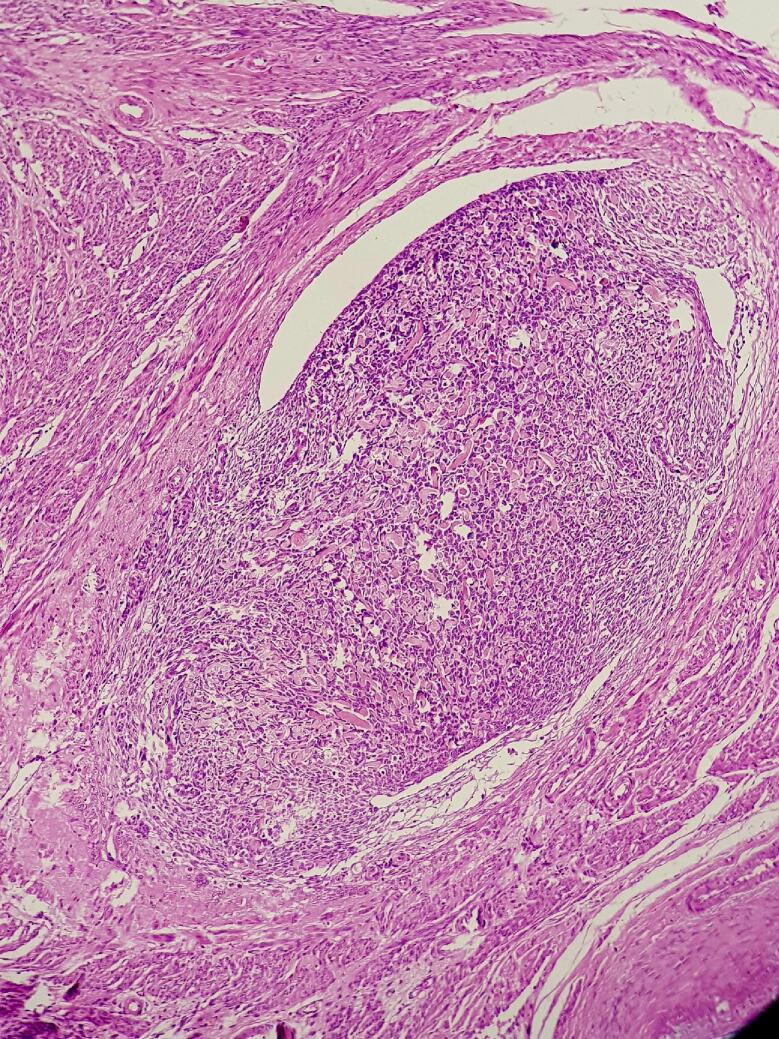
Microscopic view of uterus demonstrates monotonous atypical oval cells with mild atypia

**Fig. 8:**
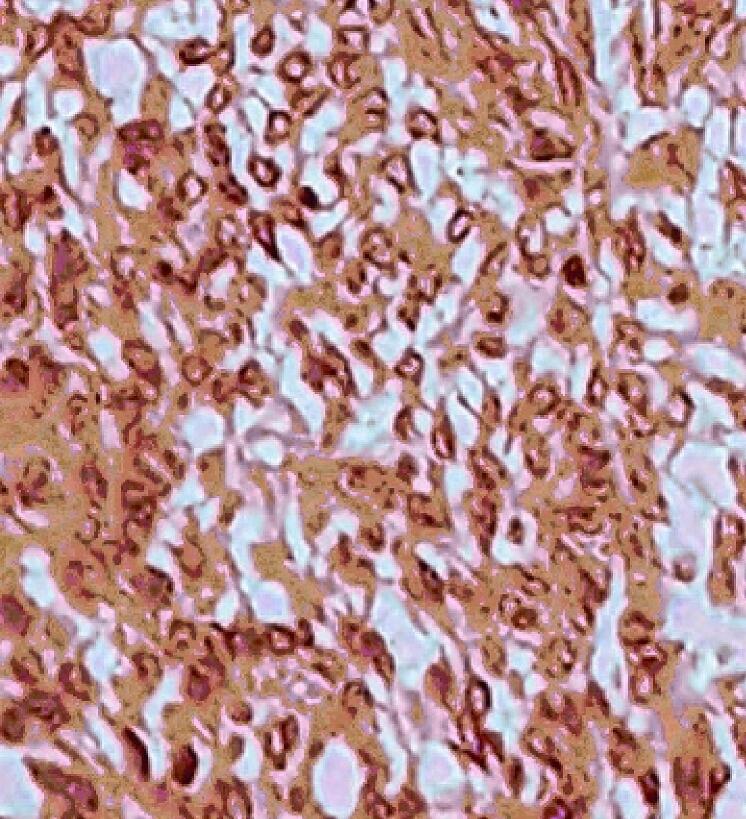
Samples are strongly positive for CD10.

An ill-defined soft whitish small polypoid lesion measuring approximately 3 cm in uterus was seen which infiltrated the myometrium in a finger like pattern. Areas of hemorrhage were also seen. Microscopically, sections revealed sheets and irregular islands of uniform tumor cells with permeative growth into the myometrium and vascular invasion in which surrounded by tumoral cells as well as low mitotic activity (<5 mitoses per 10 high power fields) (Fig. 6). These were monotonous atypical oval cells with mild atypia and might be admixed with few collagen bands. (Fig. 7). It had >3 infiltrative pattern, finger-like projections into the myometrium that each one was at least 3 mm. Immunohistochemistry stains (IHC) were applied to confirm the diagnosis besides rolling out other differential diagnosis. The samples showed high positive result for CD10 (Fig. 8), ER, and PR, whereas SMA and Inhibin, CK, Melan, CD99, and Cyclin D1 were negative in all of the tissue sections. Based on International Federation of Gynecology and Obstetrics classification [1], the final diagnosis was primary uterus LG-ESS with bilateral ovarian metastasis (stage IIA). The greater omentum, the appendix and lymph nodes were free of tumor cells.

Regarding her follow-up, hormonal therapy with letrozole 2.5 mg daily was initiated. During her 2-year follow-up, no recurrence or further post-op complications were detected. Our study has been reported in line with the SCARE criteria [9].

## 3 Discussion

LG-ESS is a rare malignant mesenchymal neoplasm consists of cells like proliferative phase of endometrial cells [10]. LG-ESS consists 0.2 % of all gynecological system malignancies with a yearly incidence of 2 per million women all over the world [11], [12]. Most common age in Low-grade endometrial stromal sarcoma (LG-ESS) is often between 42 and 58 years old. Its clinical presentation varies [10]. Obesity, diabetes mellitus and long exposure with hormones such as early menarche, late menopause and hormonal treatment are reported to be LG-ESS risk factors [8]. It has been reported that prolonged hormonal treatment such as tamoxifen or pelvic radiation are the most significant risk factors [11]. Most patients with ESS complain of pelvic discomfort, unusual vaginal bleeding as well as uterine enlargement and in less common cases due to the site of tumor vomiting, diarrhea, constipation, hematuria, increased urinary frequency, urgency, and incontinence are seen [3], [11], [13], [14]. Local relapses (i.e., pelvic or vaginal) and/or distant metastasis (e.g., abdominal wall, lungs) do occur. Few cases reported metastasis of LG-ESS to both ovaries simultaneously [14]. Some cases due to involvement of other organs or the tumor pressure effect suffer from thromboembolism besides urinary retention, urinary frequency, and urgency [8]. Imaging like ultrasound, CT (computed tomography) and MRI (magnetic resonance imaging) aren't specific For LG-ESS [10], [15]. The differential diagnosis of LG-ESS includes HG-ESS, Endometrial polyp, adenomyosis, ESN, highly cellular leiomyoma, epithelioid leiomyosarcoma, perivascular epithelioid cell tumor (PECOMA), undifferentiated carcinoma, Mixed epithelial and mesenchymal tumors (carcinosarcoma and adenosarcoma), uterine tumors resembling ovarian sex-cord tumor (UTROSCT), gland-poor adenomyosis, intravascular leiomyomata, leiomyosarcoma with extensive intravascular component, and myopericytomas (MPC) [1], [8], [16], [17]. It is clear that the diagnosis of this lesion is difficult in biopsy and curettage [18]. So, it really depends on the total extension of tumor. In some patients, LG-ESS shows different appearance due to presence of histocytes, hyalinization, atypical cells, myxoid and fibro myxoid changes, adipocytic metaplasia, rhabdoid cells, multinucleated osteoclast like cells [6]. It often causes difficulty in the initial pathological diagnosis, and the initial diagnosis is correct in only 50 % of cases [12]. Most cases of LG-ESS (60 %) present with FIGO (International Federation of Gynecology and Obstetrics classification) stage I disease and only 20 % presenting with stage IV, metastatic disease. Staging is the most important prognostic factor, with stage I and II tumors having a 5-year survival rate exceeding 90 %. On the contrary, advanced tumors have a 5-year survival rate of 40 to 50 % [2], [19], [20], [21]. Moreover, a cohort study in 2014 reported that stage, presentation of ER/PR, and nodal invasion considerably affected the LG-ESS prognosis [21]. On the contrary, a study in 2020 on LG-ESS patients introduced a new prognostic nomogram. Age, marital status, tumor size, tumor stage, chemotherapy, radiotherapy, and lymphadenectomy were evaluated in such nomogram. Age, as the most crucial prognostic factor, chemotherapy, tumor size, and tumor stage had the highest impact on LG-ESS prognosis, respectively [7].

Grossly, LG-ESS tumor size is variable between 1 and 25 cm, with a mean size of 8–11 cm. It has mostly nodular appearance with ill-defined borders that infiltrated into the myometrium, with yellowish to white cut surface, and cystic changes, hemorrhage or necrosis in some cases [12]. The histopathologic finding to differentiate LG-ESS from ESN is, myometrial invasion and lympho-vascular invasion [6]. EST with limited infiltration (EST-LI) is a pathologic finding with unknown clinical outcome, that shows myometrial invasion not as much as LG-ESS but more than in ESN (myometrial infiltration >3 mm) [6]. LG-ESS may occur outside the uterus, without any endometrial involvement related to endometriosis. For diagnosis, a wide evaluation should be done by evaluation of morphology, immunohistochemical markers, molecular study, pathology, and clinical outcome [12]. Immunohistochemistry guidance is essential to avoid misdiagnosis and making informed clinical decisions and finding of proper treatment for patients [12]. Considering the lack of specific marker for ESS, CD10 can be useful in differentiating LG-ESS from some smooth muscle tumors like leiomyosarcoma and highly cellular leiomyomas. Nonetheless, CD10, WT-1, vimentin, actins, Interferon Induced Transmembrane Protein 1 (IFITM1), estrogen, androgen, and progesterone receptors are usually positive [19], [22], [23]. The most important differential diagnosis with ESS is ESN. ESN is a benign lesion whereas LG-ESS is a sarcoma. Curettage specimens aren't sufficient to differentiate LG-ESS from ESN because both of them have analogous morphologic, immunohistochemical and molecular features. So, diagnosis is based on tumor invasion into the myometrium (myo-invasive growth pattern) and lympho-vessels [10]. Abundant chromosomal translocations are known in LG-ESS. However, approximately one third of these tumors do not harbor genetic fusions. JAZF1-SUZ12 is the most common gene fusion seen in nearly one out of two cases [6].

In regard to controversial data related to the treatment of LG-ESS, hysterectomy with bilateral salpingo-oopherectomy is a globally accepted treatment for ESS, since patients with only hysterectomy showed more recurrences [11] even in reproductive patients with LG-ESS [24]. On the other hand, some studies showed that keeping ovaries had no notable impact on the overall outcome and prognosis of patients [13], [25], [26]. Additionally, another study focused on preservation of ovaries in a young patient with LG-ESS to preserve fertility and adjuvant chemotherapy and hormonal treatment. During their 86 months follow-up, no evidence of recurrence was seen in their patient [1]. Also, one study illustrated that two patients with vaginal ESS and ovarian preservation didn't have any recurrences during their follow-ups [23], [25]. There are no strong supportive evidence that lymphadenectomy affect the prognosis and survival of LG-ESS patients. As a result, systematic or abdomino-pelvic lymphadenectomy is not recommended generally in surgical intervention of LG-ESS [8], [17].

Several reports highlighted the importance of hormonal therapy in the management of ESS tumors. Patients effectively benefit from hormonal treatment with progestinal agents, aromatase inhibitors, and gonadotropin releasing hormone (GnRH) due to expression of hormonal receptor, estrogen receptor (ER), and progesterone receptors (PR) [17], [27]. Hormonal therapy has also improved recurrence rates. Anastrozole improved prognosis significantly in a 34-year-old woman with recurrent low-grade ESS [28]. In addition, another study reported a low grade ESS in a 74-year–old woman that hormonal therapy made a considerable impact in shrinkage of the tumor before surgical removal [29]. Considering metastasized ESS, Estrogen exclusion can be very effective [11]. Two cases of LG-ESS and lung metastasis [30] and a case of a massive pelvic mass and post-renal kidney responded greatly to adjuvant therapy of Letrozole [31]. Chemotherapy is not very beneficial due to low mitotic activity [32] although responsiveness to chemotherapy was described in a reported case, suggesting total remission of ESS in combining chemotherapeutic agents [33], [34]. In spite of some positive impact of radiotherapy on HG-ESS, it has no considerable effect on low-grade forms of ESS [32], [35].

## 4 Conclusion

LG-ESS consists of a wide spectrum of differentiation and changes such as sex cord-like and smooth muscle-like differentiation. It is a challenging task to distinguish it from other subtypes of ESS. Our patient was primarily evaluated due to large ovarian mass. During our pathological investigation, incidental uterus LG-ESS with bilateral ovarian invasion was confirmed who was successfully treated by surgical intervention and hormonal therapy. Due to controversial data regarding LG-ESS diagnosis, prognosis, and treatment, further studies are required to ease the path to a definite diagnosis and treatment of LG-ESS patients with metastasis.

## Consent for publication

Written informed consent was obtained from the patient for publication of this case report and any accompanying images. A copy of the written consent is available for review by the Editor-in-Chief of this journal.

## Ethical approval

Not applicable.

## Funding

This study received no funding.

## Guarantor

Dr Mahsa Salehi.

## CRediT authorship contribution statement

Dr Sheidaei and Salehi provided the main idea behind this case study and reviewed and approved the written article. Dr Afrooz Jafari participated in gathering data and assisted in the patients' surgery. Dr Hamid Reza Jafari and Salehi wrote the manuscript and did the final revisions as well as subscribing the article.

## Competing interest

There is no conflict of interest.

## Data Availability

Not applicable.
